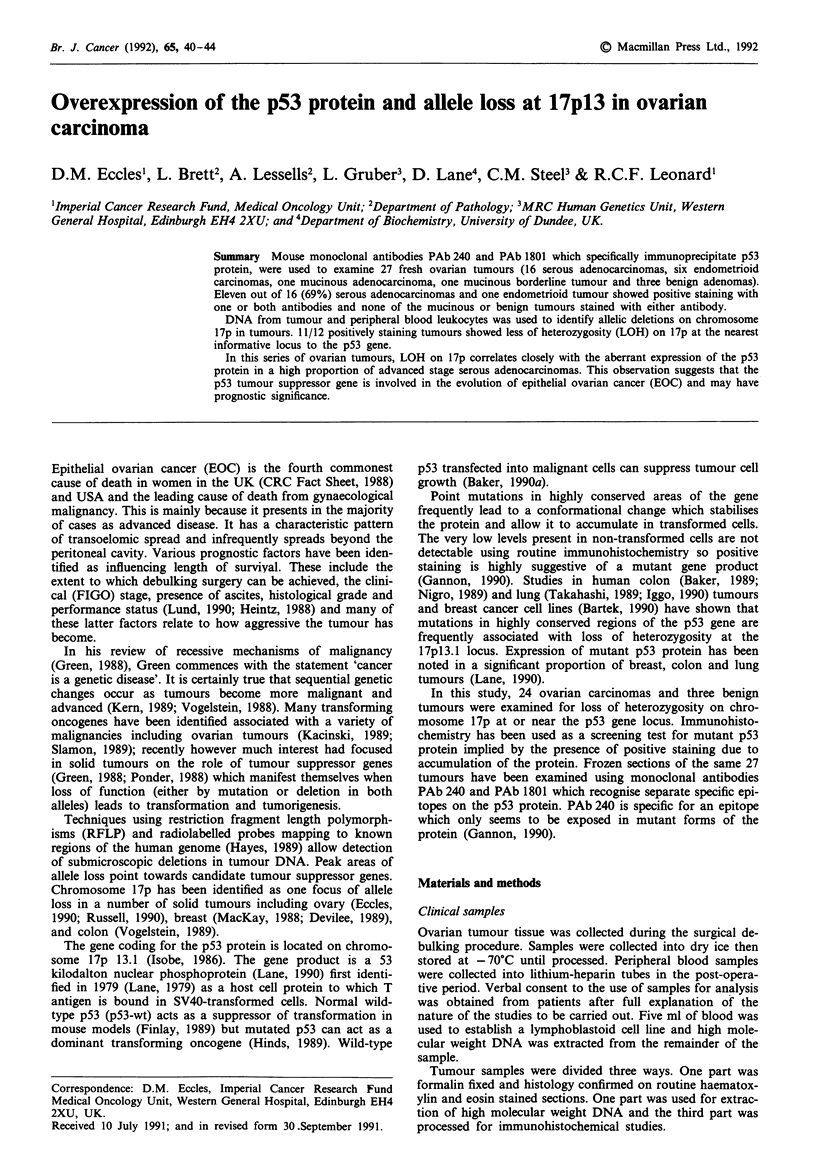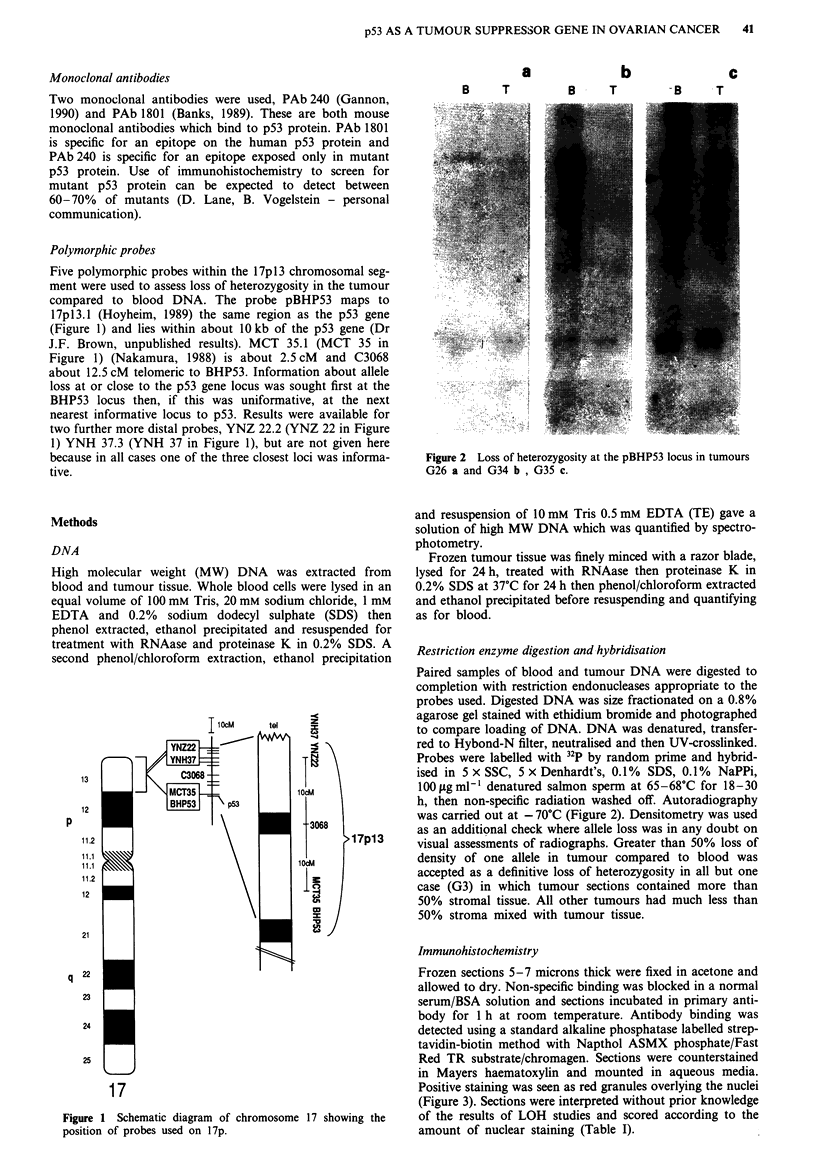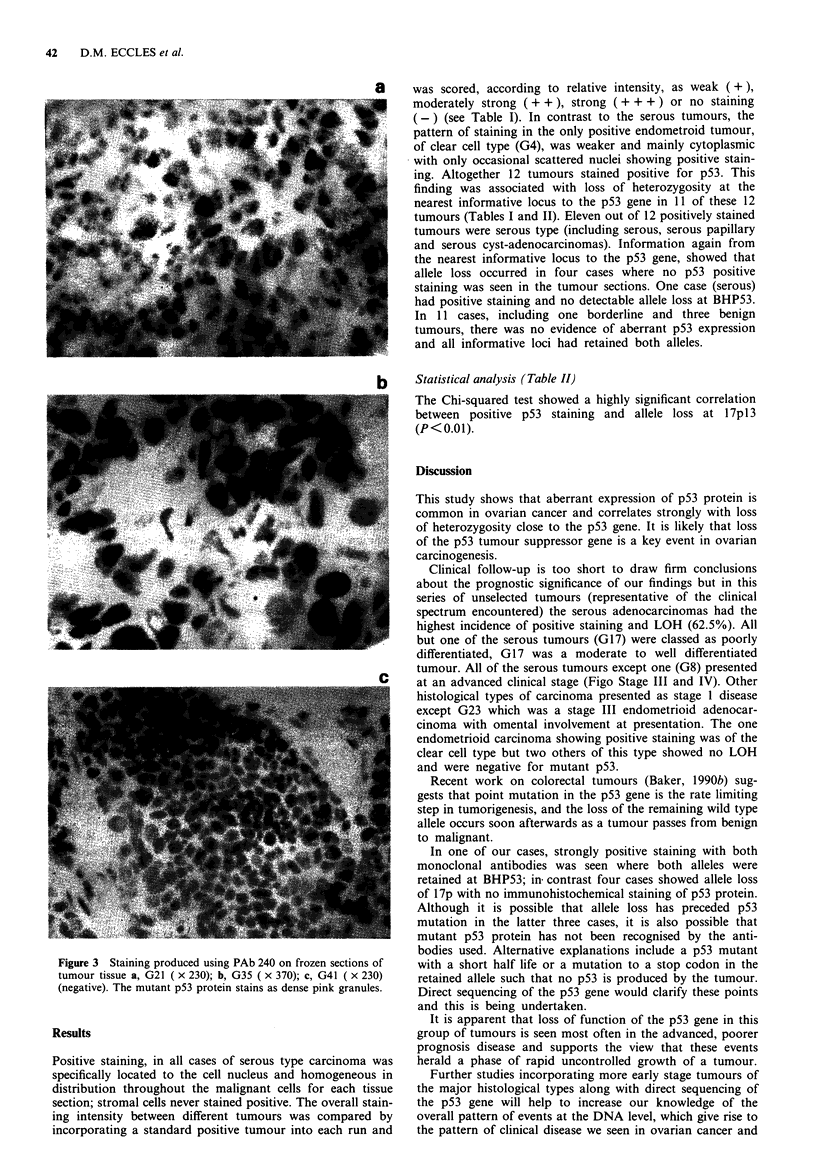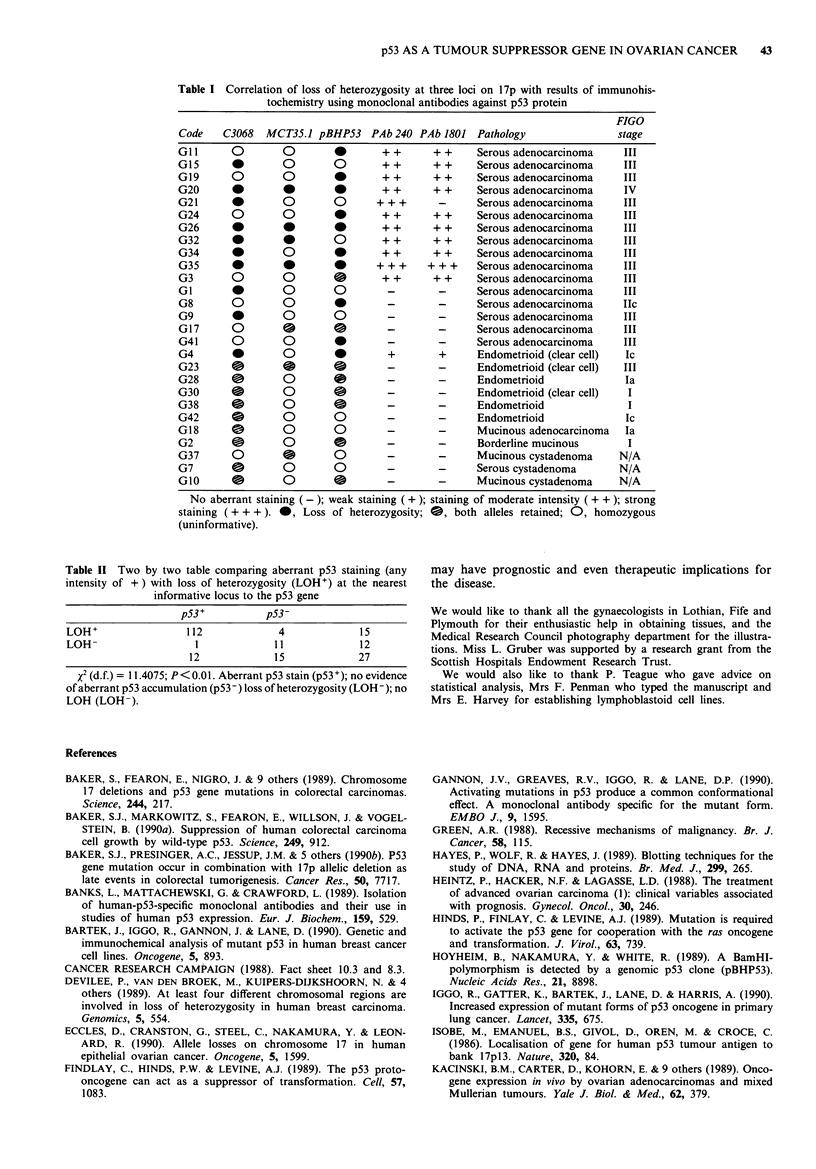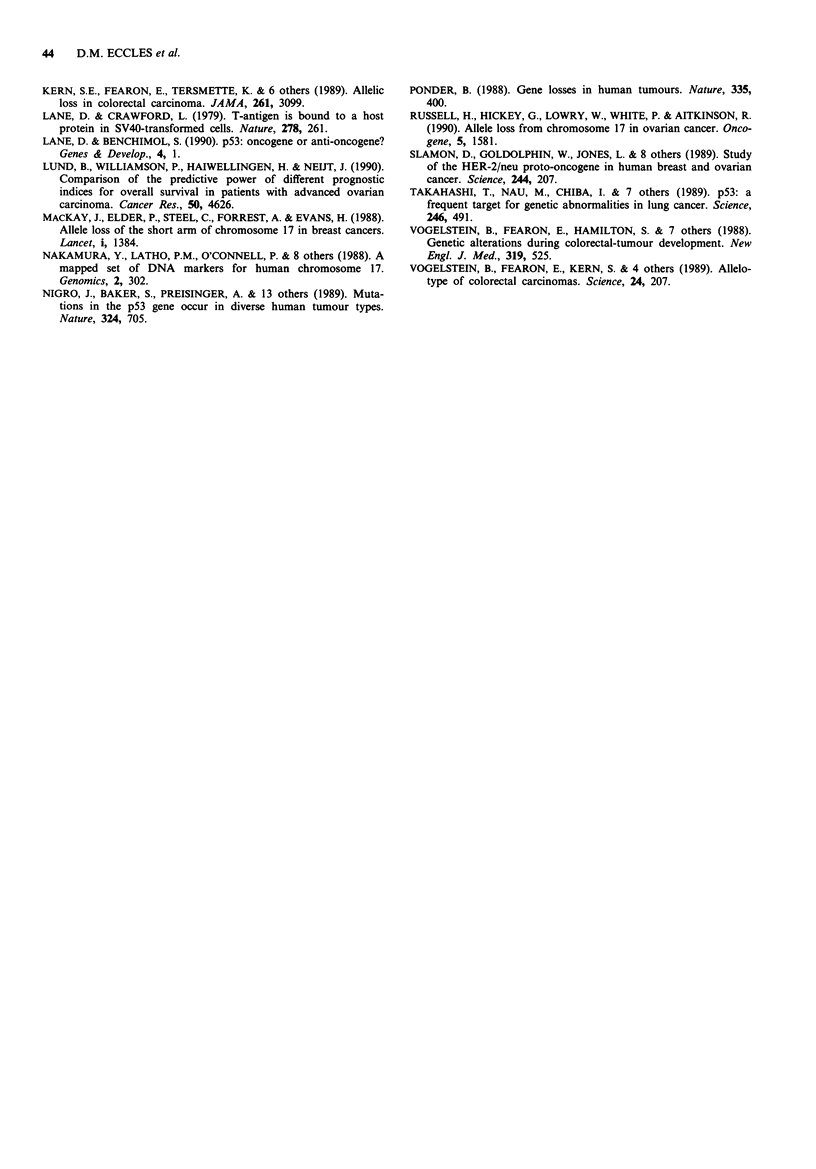# Overexpression of the p53 protein and allele loss at 17p13 in ovarian carcinoma.

**DOI:** 10.1038/bjc.1992.8

**Published:** 1992-01

**Authors:** D. M. Eccles, L. Brett, A. Lessells, L. Gruber, D. Lane, C. M. Steel, R. C. Leonard

**Affiliations:** Imperial Cancer Research Fund, Medical Oncology Unit, Western General Hospital, Edinburgh, UK.

## Abstract

**Images:**


Br. J. Cancer (1992), 65, 40–44 © Macmillan Press Ltd., 1992

### Overexpression of the p53 protein and allele loss at 17p13 in ovarian carcinoma

D.M. Eccles[1], L. Brett[2], A. Lessells[2], L. Gruber[3], D. Lane[4], C.M. Steel[3] & R.C.F. Leonard[1]

[1]*Imperial Cancer Research Fund, Medical Oncology Unit;* [2]*Department of Pathology;* [3]*MRC Human Genetics Unit, Western General Hospital, Edinburgh EH4 2XU; and* [4]*Department of Biochemistry, University of Dundee, UK.*

**Summary** Mouse monoclonal antibodies PAb 240 and PAb 1801 which specifically immunoprecipitate p53 protein, were used to examine 27 fresh ovarian tumours (16 serous adenocarcinomas, six endometrioid carcinomas, one mucinous adenocarcinoma, one mucinous borderline tumour and three benign adenomas). Eleven out of 16 (69%) serous adenocarcinomas and one endometrioid tumour showed positive staining with one or both antibodies and none of the mucinous or benign tumours stained with either antibody.

DNA from tumour and peripheral blood leukocytes was used to identify allelic deletions on chromosome 17p in tumours. 11/12 positively staining tumours showed less of heterozygosity (LOH) on 17p at the nearest informative locus to the p53 gene.

In this series of ovarian tumours, LOH on 17p correlates closely with the aberrant expression of the p53 protein in a high proportion of advanced stage serous adenocarcinomas. This observation suggests that the p53 tumour suppressor gene is involved in the evolution of epithelial ovarian cancer (EOC) and may have prognostic significance.

Epithelial ovarian cancer (EOC) is the fourth commonest cause of death in women in the UK (CRC Fact Sheet, 1988) and USA and the leading cause of death from gynaecological malignancy. This is mainly because it presents in the majority of cases as advanced disease. It has a characteristic pattern of transoelomic spread and infrequently spreads beyond the peritoneal cavity. Various prognostic factors have been identified as influencing length of surviyal. These include the extent to which debulking surgery can be achieved, the clinical (FIGO) stage, presence of ascites, histological grade and performance status (Lund, 1990; Heintz, 1988) and many of these latter factors relate to how aggressive the tumour has become.

In his review of recessive mechanisms of malignancy (Green, 1988), Green commences with the statement 'cancer is a genetic disease'. It is certainly true that sequential genetic changes occur as tumours become more malignant and advanced (Kern, 1989; Vogelstein, 1988). Many transforming oncogenes have been identified associated with a variety of malignancies including ovarian tumours (Kacinski, 1989; Slamon, 1989); recently however much interest had focused in solid tumours on the role of tumour suppressor genes (Green, 1988; Ponder, 1988) which manifest themselves when loss of function (either by mutation or deletion in both alleles) leads to transformation and tumorigenesis.

Techniques using restriction fragment length polymorphisms (RFLP) and radiolabelled probes mapping to known regions of the human genome (Hayes, 1989) allow detection of submicroscopic deletions in tumour DNA. Peak areas of allele loss point towards candidate tumour suppressor genes. Chromosome 17p has been identified as one focus of allele loss in a number of solid tumours including ovary (Eccles, 1990; Russell, 1990), breast (MacKay, 1988; Devilee, 1989), and colon (Vogelstein, 1989).

The gene coding for the p53 protein is located on chromosome 17p 13.1 (Isobe, 1986). The gene product is a 53 kilodalton nuclear phosphoprotein (Lane, 1990) first identified in 1979 (Lane, 1979) as a host cell protein to which T antigen is bound in SV40-transformed cells. Normal wild-type p53 (p53-wt) acts as a suppressor of transformation in mouse models (Finlay, 1989) but mutated p53 can act as a dominant transforming oncogene (Hinds, 1989). Wild-type p53 transfected into malignant cells can suppress tumour cell growth (Baker, 1990*a*).

Point mutations in highly conserved areas of the gene frequently lead to a conformational change which stabilises the protein and allow it to accumulate in transformed cells. The very low levels present in non-transformed cells are not detectable using routine immunohistochemistry so positive staining is highly suggestive of a mutant gene product (Gannon, 1990). Studies in human colon (Baker, 1989; Nigro, 1989) and lung (Takahashi, 1989; Iggo, 1990) tumours and breast cancer cell lines (Bartek, 1990) have shown that mutations in highly conserved regions of the p53 gene are frequently associated with loss of heterozygosity at the 17p13.1 locus. Expression of mutant p53 protein has been noted in a significant proportion of breast, colon and lung tumours (Lane, 1990).

In this study, 24 ovarian carcinomas and three benign tumours were examined for loss of heterozygosity on chromosome 17p at or near the p53 gene locus. Immunohistochemistry has been used as a screening test for mutant p53 protein implied by the presence of positive staining due to accumulation of the protein. Frozen sections of the same 27 tumours have been examined using monoclonal antibodies PAb 240 and PAb 1801 which recognise separate specific epitopes on the p53 protein. PAb 240 is specific for an epitope which only seems to be exposed in mutant forms of the protein (Gannon, 1990).

### Materials and methods

### *Clinical samples*

Ovarian tumour tissue was collected during the surgical debulking procedure. Samples were collected into dry ice then stored at −70°C until processed. Peripheral blood samples were collected into lithium-heparin tubes in the post-operative period. Verbal consent to the use of samples for analysis was obtained from patients after full explanation of the nature of the studies to be carried out. Five ml of blood was used to establish a lymphoblastoid cell line and high molecular weight DNA was extracted from the remainder of the sample.

Tumour samples were divided three ways. One part was formalin fixed and histology confirmed on routine haematoxylin and eosin stained sections. One part was used for extraction of high molecular weight DNA and the third part was processed for immunohistochemical studies.

Correspondence: D.M. Eccles, Imperial Cancer Research Fund Medical Oncology Unit, Western General Hospital, Edinburgh EH4 2XU, UK.
Received 10 July 1991; and in revised form 30 September 1991.

*Monoclonal antibodies*

Two monoclonal antibodies were used, PAb 240 (Gannon, 1990) and PAb 1801 (Banks, 1989). These are both mouse monoclonal antibodies which bind to p53 protein. PAb 1801 is specific for an epitope on the human p53 protein and PAb 240 is specific for an epitope exposed only in mutant p53 protein. Use of immunohistochemistry to screen for mutant p53 protein can be expected to detect between 60–70% of mutants (D. Lane, B. Vogelstein – personal communication).

*Polymorphic probes*

Five polymorphic probes within the 17p13 chromosomal segment were used to assess loss of heterozygosity in the tumour compared to blood DNA. The probe pBHP53 maps to 17p13.1 (Hoyheim, 1989) the same region as the p53 gene (Figure 1) and lies within about 10 kb of the p53 gene (Dr J.F. Brown, unpublished results). MCT 35.1 (MCT 35 in Figure 1) (Nakamura, 1988) is about 2.5 cM and C3068 about 12.5 cM telomeric to BHP53. Information about allele loss at or close to the p53 gene locus was sought first at the BHP53 locus then, if this was uniformative, at the next nearest informative locus to p53. Results were available for two further more distal probes, YNZ 22.2 (YNZ 22 in Figure 1) YNH 37.3 (YNH 37 in Figure 1), but are not given here because in all cases one of the three closest loci was informative.

**Figure 2** Loss of heterozygosity at the pBHP53 locus in tumours G26 **a** and G34 **b** , G35 **c**.

### Methods

*DNA*

High molecular weight (MW) DNA was extracted from blood and tumour tissue. Whole blood cells were lysed in an equal volume of 100 mM Tris, 20 mM sodium chloride, 1 mM EDTA and 0.2% sodium dodecyl sulphate (SDS) then phenol extracted, ethanol precipitated and resuspended for treatment with RNAase and proteinase K in 0.2% SDS. A second phenol/chloroform extraction, ethanol precipitation and resuspension of 10 mM Tris 0.5 mM EDTA (TE) gave a solution of high MW DNA which was quantified by spectrophotometry.

Frozen tumour tissue was finely minced with a razor blade, lysed for 24 h, treated with RNAase then proteinase K in 0.2% SDS at 37°C for 24 h then phenol/chloroform extracted and ethanol precipitated before resuspending and quantifying as for blood.

**Figure 1** Schematic diagram of chromosome 17 showing the position of probes used on 17p.

*Restriction enzyme digestion and hybridisation*

Paired samples of blood and tumour DNA were digested to completion with restriction endonucleases appropriate to the probes used. Digested DNA was size fractionated on a 0.8% agarose gel stained with ethidium bromide and photographed to compare loading of DNA. DNA was denatured, transferred to Hybond-N filter, neutralised and then UV-crosslinked. Probes were labelled with $^{32}P$ by random prime and hybridised in 5 × SSC, 5 × Denhardt's, 0.1% SDS, 0.1% NaPPi, 100 $\mu g\ ml^{-1}$ denatured salmon sperm at 65–68°C for 18–30 h, then non-specific radiation washed off. Autoradiography was carried out at −70°C (Figure 2). Densitometry was used as an additional check where allele loss was in any doubt on visual assessments of radiographs. Greater than 50% loss of density of one allele in tumour compared to blood was accepted as a definitive loss of heterozygosity in all but one case (G3) in which tumour sections contained more than 50% stromal tissue. All other tumours had much less than 50% stroma mixed with tumour tissue.

*Immunohistochemistry*

Frozen sections 5–7 microns thick were fixed in acetone and allowed to dry. Non-specific binding was blocked in a normal serum/BSA solution and sections incubated in primary antibody for 1 h at room temperature. Antibody binding was detected using a standard alkaline phosphatase labelled streptavidin-biotin method with Napthol ASMX phosphate/Fast Red TR substrate/chromagen. Sections were counterstained in Mayers haematoxylin and mounted in aqueous media. Positive staining was seen as red granules overlying the nuclei (Figure 3). Sections were interpreted without prior knowledge of the results of LOH studies and scored according to the amount of nuclear staining (Table I).

**Figure 3** Staining produced using PAb 240 on frozen sections of tumour tissue **a**, G21 ( × 230); **b**, G35 ( × 370); **c**, G41 ( × 230) (negative). The mutant p53 protein stains as dense pink granules.

### Results

Positive staining, in all cases of serous type carcinoma was specifically located to the cell nucleus and homogeneous in distribution throughout the malignant cells for each tissue section; stromal cells never stained positive. The overall staining intensity between different tumours was compared by incorporating a standard positive tumour into each run and was scored, according to relative intensity, as weak ( + ), moderately strong ( + + ), strong ( + + + ) or no staining ( – ) (see Table I). In contrast to the serous tumours, the pattern of staining in the only positive endometroid tumour, of clear cell type (G4), was weaker and mainly cytoplasmic with only occasional scattered nuclei showing positive staining. Altogether 12 tumours stained positive for p53. This finding was associated with loss of heterozygosity at the nearest informative locus to the p53 gene in 11 of these 12 tumours (Tables I and II). Eleven out of 12 positively stained tumours were serous type (including serous, serous papillary and serous cyst-adenocarcinomas). Information again from the nearest informative locus to the p53 gene, showed that allele loss occurred in four cases where no p53 positive staining was seen in the tumour sections. One case (serous) had positive staining and no detectable allele loss at BHP53. In 11 cases, including one borderline and three benign tumours, there was no evidence of aberrant p53 expression and all informative loci had retained both alleles.

### *Statistical analysis (Table II)*

The Chi-squared test showed a highly significant correlation between positive p53 staining and allele loss at 17p13 ($P < 0.01$).

### Discussion

This study shows that aberrant expression of p53 protein is common in ovarian cancer and correlates strongly with loss of heterozygosity close to the p53 gene. It is likely that loss of the p53 tumour suppressor gene is a key event in ovarian carcinogenesis.

Clinical follow-up is too short to draw firm conclusions about the prognostic significance of our findings but in this series of unselected tumours (representative of the clinical spectrum encountered) the serous adenocarcinomas had the highest incidence of positive staining and LOH (62.5%). All but one of the serous tumours (G17) were classed as poorly differentiated, G17 was a moderate to well differentiated tumour. All of the serous tumours except one (G8) presented at an advanced clinical stage (Figo Stage III and IV). Other histological types of carcinoma presented as stage 1 disease except G23 which was a stage III endometrioid adenocarcinoma with omental involvement at presentation. The one endometrioid carcinoma showing positive staining was of the clear cell type but two others of this type showed no LOH and were negative for mutant p53.

Recent work on colorectal tumours (Baker, 1990*b*) suggests that point mutation in the p53 gene is the rate limiting step in tumorigenesis, and the loss of the remaining wild type allele occurs soon afterwards as a tumour passes from benign to malignant.

In one of our cases, strongly positive staining with both monoclonal antibodies was seen where both alleles were retained at BHP53; in contrast four cases showed allele loss of 17p with no immunohistochemical staining of p53 protein. Although it is possible that allele loss has preceded p53 mutation in the latter three cases, it is also possible that mutant p53 protein has not been recognised by the antibodies used. Alternative explanations include a p53 mutant with a short half life or a mutation to a stop codon in the retained allele such that no p53 is produced by the tumour. Direct sequencing of the p53 gene would clarify these points and this is being undertaken.

It is apparent that loss of function of the p53 gene in this group of tumours is seen most often in the advanced, poorer prognosis disease and supports the view that these events herald a phase of rapid uncontrolled growth of a tumour.

Further studies incorporating more early stage tumours of the major histological types along with direct sequencing of the p53 gene will help to increase our knowledge of the overall pattern of events at the DNA level, which give rise to the pattern of clinical disease we seen in ovarian cancer and

**Table I** Correlation of loss of heterozygosity at three loci on 17p with results of immunohistochemistry using monoclonal antibodies against p53 protein

| *Code* | *C3068* | *MCT35.1* | *pBHP53* | *PAb 240* | *PAb 1801* | *Pathology* | *FIGO stage* |
|---|---|---|---|---|---|---|---|
| G11 | ○ | ○ | ● | ++ | ++ | Serous adenocarcinoma | III |
| G15 | ● | ○ | ○ | ++ | ++ | Serous adenocarcinoma | III |
| G19 | ○ | ○ | ● | ++ | ++ | Serous adenocarcinoma | III |
| G20 | ● | ● | ● | ++ | ++ | Serous adenocarcinoma | IV |
| G21 | ● | ○ | ○ | +++ | – | Serous adenocarcinoma | III |
| G24 | ○ | ○ | ● | ++ | ++ | Serous adenocarcinoma | III |
| G26 | ● | ● | ● | ++ | ++ | Serous adenocarcinoma | III |
| G32 | ● | ● | ○ | ++ | ++ | Serous adenocarcinoma | III |
| G34 | ● | ○ | ● | ++ | ++ | Serous adenocarcinoma | III |
| G35 | ● | ● | ● | +++ | +++ | Serous adenocarcinoma | III |
| G3 | ○ | ○ | ⊘ | ++ | ++ | Serous adenocarcinoma | III |
| G1 | ● | ○ | ○ | – | – | Serous adenocarcinoma | III |
| G8 | ○ | ○ | ● | – | – | Serous adenocarcinoma | IIc |
| G9 | ● | ○ | ○ | – | – | Serous adenocarcinoma | III |
| G17 | ○ | ⊘ | ⊘ | – | – | Serous adenocarcinoma | III |
| G41 | ○ | ○ | ● | – | – | Serous adenocarcinoma | III |
| G4 | ● | ○ | ● | + | + | Endometrioid (clear cell) | Ic |
| G23 | ⊘ | ⊘ | ⊘ | – | – | Endometrioid (clear cell) | III |
| G28 | ⊘ | ○ | ⊘ | – | – | Endometrioid | Ia |
| G30 | ⊘ | ○ | ⊘ | – | – | Endometrioid (clear cell) | I |
| G38 | ⊘ | ○ | ⊘ | – | – | Endometrioid | I |
| G42 | ⊘ | ○ | ○ | – | – | Endometrioid | Ic |
| G18 | ⊘ | ○ | ○ | – | – | Mucinous adenocarcinoma | Ia |
| G2 | ⊘ | ○ | ⊘ | – | – | Borderline mucinous | I |
| G37 | ○ | ⊘ | ○ | – | – | Mucinous cystadenoma | N/A |
| G7 | ⊘ | ○ | ○ | – | – | Serous cystadenoma | N/A |
| G10 | ⊘ | ○ | ⊘ | – | – | Mucinous cystadenoma | N/A |

No aberrant staining ( – ); weak staining ( + ); staining of moderate intensity ( + + ); strong staining ( + + + ). ●, Loss of heterozygosity; ⊘, both alleles retained; ○, homozygous (uninformative).

**Table II** Two by two table comparing aberrant p53 staining (any intensity of + ) with loss of heterozygosity ($LOH^+$) at the nearest informative locus to the p53 gene

| | $p53^+$ | $p53^-$ | |
|---|---|---|---|
| $LOH^+$ | 112 | 4 | 15 |
| $LOH^-$ | 1 | 11 | 12 |
| | 12 | 15 | 27 |

$\chi^2$ (d.f.) = 11.4075; $P < 0.01$. Aberrant p53 stain ($p53^+$); no evidence of aberrant p53 accumulation ($p53^-$) loss of heterozygosity ($LOH^-$); no LOH ($LOH^-$).

may have prognostic and even therapeutic implications for the disease.

We would like to thank all the gynaecologists in Lothian, Fife and Plymouth for their enthusiastic help in obtaining tissues, and the Medical Research Council photography department for the illustrations. Miss L. Gruber was supported by a research grant from the Scottish Hospitals Endowment Research Trust.

We would also like to thank P. Teague who gave advice on statistical analysis, Mrs F. Penman who typed the manuscript and Mrs E. Harvey for establishing lymphoblastoid cell lines.

### References

BAKER, S., FEARON, E., NIGRO, J. & 9 others (1989). Chromosome 17 deletions and p53 gene mutations in colorectal carcinomas. *Science*, **244**, 217.

BAKER, S.J., MARKOWITZ, S., FEARON, E., WILLSON, J. & VOGELSTEIN, B. (1990*a*). Suppression of human colorectal carcinoma cell growth by wild-type p53. *Science*, **249**, 912.

BAKER, S.J., PRESINGER, A.C., JESSUP, J.M. & 5 others (1990*b*). P53 gene mutation occur in combination with 17p allelic deletion as late events in colorectal tumorigenesis. *Cancer Res.*, **50**, 7717.

BANKS, L., MATTACHEWSKI, G. & CRAWFORD, L. (1989). Isolation of human-p53-specific monoclonal antibodies and their use in studies of human p53 expression. *Eur. J. Biochem.*, **159**, 529.

BARTEK, J., IGGO, R., GANNON, J. & LANE, D. (1990). Genetic and immunochemical analysis of mutant p53 in human breast cancer cell lines. *Oncogene*, **5**, 893.

CANCER RESEARCH CAMPAIGN (1988). Fact sheet 10.3 and 8.3.

DEVILEE, P., VAN DEN BROEK, M., KUIPERS-DIJKSHOORN, N. & 4 others (1989). At least four different chromosomal regions are involved in loss of heterozygosity in human breast carcinoma. *Genomics*, **5**, 554.

ECCLES, D., CRANSTON, G., STEEL, C., NAKAMURA, Y. & LEONARD, R. (1990). Allele losses on chromosome 17 in human epithelial ovarian cancer. *Oncogene*, **5**, 1599.

FINDLAY, C., HINDS, P.W. & LEVINE, A.J. (1989). The p53 protooncogene can act as a suppressor of transformation. *Cell*, **57**, 1083.

GANNON, J.V., GREAVES, R.V., IGGO, R. & LANE, D.P. (1990). Activating mutations in p53 produce a common conformational effect. A monoclonal antibody specific for the mutant form. *EMBO J.*, **9**, 1595.

GREEN, A.R. (1988). Recessive mechanisms of malignancy. *Br. J. Cancer*, **58**, 115.

HAYES, P., WOLF, R. & HAYES, J. (1989). Blotting techniques for the study of DNA, RNA and proteins. *Br. Med. J.*, **299**, 265.

HEINTZ, P., HACKER, N.F. & LAGASSE, L.D. (1988). The treatment of advanced ovarian carcinoma (1): clinical variables associated with prognosis. *Gynecol. Oncol.*, **30**, 246.

HINDS, P., FINLAY, C. & LEVINE, A.J. (1989). Mutation is required to activate the p53 gene for cooperation with the *ras* oncogene and transformation. *J. Virol.*, **63**, 739.

HOYHEIM, B., NAKAMURA, Y. & WHITE, R. (1989). A BamHI-polymorphism is detected by a genomic p53 clone (pBHP53). *Nucleic Acids Res.*, **21**, 8898.

IGGO, R., GATTER, K., BARTEK, J., LANE, D. & HARRIS, A. (1990). Increased expression of mutant forms of p53 oncogene in primary lung cancer. *Lancet*, **335**, 675.

ISOBE, M., EMANUEL, B.S., GIVOL, D., OREN, M. & CROCE, C. (1986). Localisation of gene for human p53 tumour antigen to bank 17p13. *Nature*, **320**, 84.

KACINSKI, B.M., CARTER, D., KOHORN, E. & 9 others (1989). Oncogene expression *in vivo* by ovarian adenocarcinomas and mixed Mullerian tumours. *Yale J. Biol. & Med.*, **62**, 379.

KERN, S.E., FEARON, E., TERSMETTE, K. & 6 others (1989). Allelic loss in colorectal carcinoma. *JAMA*, **261**, 3099.

LANE, D. & CRAWFORD, L. (1979). T-antigen is bound to a host protein in SV40-transformed cells. *Nature*, **278**, 261.

LANE, D. & BENCHIMOL, S. (1990). p53: oncogene or anti-oncogene? *Genes & Develop.*, **4**, 1.

LUND, B., WILLIAMSON, P., HAIWELLINGEN, H. & NEIJT, J. (1990). Comparison of the predictive power of different prognostic indices for overall survival in patients with advanced ovarian carcinoma. *Cancer Res.*, **50**, 4626.

MACKAY, J., ELDER, P., STEEL, C., FORREST, A. & EVANS, H. (1988). Allele loss of the short arm of chromosome 17 in breast cancers. *Lancet*, **i**, 1384.

NAKAMURA, Y., LATHO, P.M., O'CONNELL, P. & 8 others (1988). A mapped set of DNA markers for human chromosome 17. *Genomics*, **2**, 302.

NIGRO, J., BAKER, S., PREISINGER, A. & 13 others (1989). Mutations in the p53 gene occur in diverse human tumour types. *Nature*, **324**, 705.

PONDER, B. (1988). Gene losses in human tumours. *Nature*, **335**, 400.

RUSSELL, H., HICKEY, G., LOWRY, W., WHITE, P. & AITKINSON, R. (1990). Allele loss from chromosome 17 in ovarian cancer. *Oncogene*, **5**, 1581.

SLAMON, D., GOLDOLPHIN, W., JONES, L. & 8 others (1989). Study of the HER-2/neu proto-oncogene in human breast and ovarian cancer. *Science*, **244**, 207.

TAKAHASHI, T., NAU, M., CHIBA, I. & 7 others (1989). p53: a frequent target for genetic abnormalities in lung cancer. *Science*, **246**, 491.

VOGELSTEIN, B., FEARON, E., HAMILTON, S. & 7 others (1988). Genetic alterations during colorectal-tumour development. *New Engl. J. Med.*, **319**, 525.

VOGELSTEIN, B., FEARON, E., KERN, S. & 4 others (1989). Allelotype of colorectal carcinomas. *Science*, **24**, 207.